# The nutriRECIPE-Index – development and validation of a nutrient-weighted index for the evaluation of recipes

**DOI:** 10.1186/s40795-021-00483-7

**Published:** 2021-11-18

**Authors:** Frank Forner, Ina Volkhardt, Toni Meier, Olaf Christen, Gabriele I. Stangl

**Affiliations:** 1 Martin-Luther-University Halle-Wittenberg, Institute for Agricultural and Nutritional Sciences, Halle, Germany; 2 NutriCARD Competence Cluster for Nutrition and Cardiovascular Health, Halle-Jena Leipzig, Leipzig, Germany

**Keywords:** Diet quality, Public catering, Healthy meal index, Nutri-score, Public health nutrition

## Abstract

**Background:**

Our objective was to develop a nutrient-based index for evaluating and improving menus in public catering. The nutriRECIPE-Index comprises 24 nutrients and nutrient groups. In developing the index, the following steps were included: setting the goals of the index, nutrient selection, target metrics and scaling, weighting, proof of concept and validation of the index. Furthermore, a unique database was created to integrate bioactive plant compounds in the assessment. An assessment of standard recipes and supposedly healthy recipes should show a significant difference in the results of the nutriRECIPE-Index. Finally, the nutriRECIPE-Index should generate similar or more specific results than existing indices such as the Nutri-Score and the Healthy Meal Index.

**Methods:**

A whole meal cycle (comprising 6 weeks, 106 recipes and including different menu lines, partially with different side dishes) at a university canteen was analysed with the Federal Food Code (BLS) and the nutriRECIPE-Index. The Healthy Meal Index (comprising 3 nutritionally relevant items) and the Nutri-Score algorithm (comprising 7 items) were used to validate the nutrient composition and the results of the nutriRECIPE-Index.

**Results:**

The resulting scores of the recipes and menu lines showed substantial differences, wherein the meals of a health-promoting menu line usually received higher scores than the standard recipes. A correlation between the nutriRECIPE-Index and the Healthy Meal Index (0.604) and the Nutri-Score (0.591) was observed. The nutriRECIPE-Index was better at identifying the worst menus and could better separate mediocre menus from good menus.

**Conclusion:**

The nutriRECIPE-Index is a useful and comprehensive tool for evaluating the nutritional value of recipes and is the first to consider bioactive plant compounds. Further adjustments to different target populations, settings, and cultural backgrounds are possible.

**Supplementary Information:**

The online version contains supplementary material available at 10.1186/s40795-021-00483-7.

## Introduction

In addition to undernourishment, an unbalanced diet, also known as malnutrition, is one of the main disease risks globally. In 2017, malnutrition led to 9.5 million premature deaths from cardiovascular diseases, 0.9 million cancer deaths and 0.5 million deaths due to diabetes and chronic kidney disease [1, 2]. In Europe, almost 25% of all premature deaths are caused by diet-related cardiovascular diseases [3]. A large part of this situation is due to the limited food offerings in the market, poor nutritional knowledge and lack of relevant information at the point of sale [4, 5]. Several empirical studies have shown that improved information on the health value of food can support consumers in their purchasing decisions [6, 7].

In 2017, 7.4 billion euros were spent in Germany on company catering – an increase of 200 million euros and therefore almost 3% over the previous year [8]. In the EU-28, the food services sector recorded a value-added of approximately EUR 175.5 million in 2016 [9]. This demonstrates the growing importance of out-of-home catering. High consumption of meals outside the home has been associated with an increased body mass index (BMI) [10, 11]. Thus, improvement in the nutritional quality of these meals could contribute to public health. Although policymakers have recognised the importance of nutrition for public health, activities to improve public nutrition are (mostly) limited to food labelling [12]. Moreover, the advertising of health-promoting services, including in canteens, is subject to legal limits within the EU [13]. However, numerous reviews have shown that measures such as recipe changes, changes in portion sizes, adjusting of prices, and more sophisticated labelling allow consumers to make more health-promoting choices [14–16].

### Scope of existing models for evaluating nutrition and menus

Currently, a large number of a priori defined models (indices) for the assessment of nutrition (and nutrition patterns) exist; these tools are food- or food group-based or nutrient-based or contain elements of both approaches [17]. They also differ in factors such as the objective of the application and scaling.

For individual menus, in particular, there are only a few models for evaluation and optimisation; examples include the Healthy Meal Index [18], the Nutri-Score [19], the susDISH method [20], NutriScale [21], the Menu Sustainability Index [22], the NAHGAST method [23] and the vegan checklist [24]. The association between the index and health outcomes is often low [25–27] or has not been explicitly evaluated.

### Bioactive plant compounds (BPCs)

Although single, compound-specific dose-outcome curves are difficult to investigate in human trials (due to the abundance and diversity of BPCs in foods, in particular in fruits and vegetables), epidemiological studies show that BPCs – in addition to macro- and micronutrients – play a major role in the primary and secondary prevention of noncommunicable diseases [28–31]. It has been shown that an increased intake of lycopene – a carotenoid found in tomatoes and carrots – had beneficial effects on blood lipids, blood pressure, and endothelial function [32]. Phytosterols – found in nuts and vegetable oils – have an LDL cholesterol-lowering effect [33]. However, BPCs are currently not integrated into existing indices evaluating the nutritional quality of food.

### Aim of the study

To overcome the limitations of existing nutrient-based evaluation models (narrow scope, arbitrary weighting of components, no explicit consideration of BPCs), the nutriRECIPE-Index was developed and tested using 106 recipes from a university canteen. Therefore, a nutrient-weighted recipe evaluation model, which considers 19 macro- and micronutrients with an official recommendation status (according to the German, Austrian and Swiss Nutrition Societies), was developed. The model included BPCs to consider the health value of bioactive plant compounds more strongly. It was validated using the HMI and Nutri-Score.

## Methods

### Model development

Following the review of Waijers et al. [25], the following steps were considered in the development of the nutriRECIPE-Index: definition of the aim of the index, choice of component type (e.g., nutrient, nutrient ratios or food groups), selection of the components (in this case various nutrients), target sizes and scaling, weighting, practical test and adjustment, and validation.

The aim of the nutriRECIPE-Index is the assessment (and thus the possible optimisation) of individual recipes for meals, considering 19 macro- and micronutrients and five BPC groups with high public health relevance. Table 1 provides an overview of the nutrients and BPC groups included. Moreover, the nutriRECIPE-Index distinguishes between moderation and adequacy components, as proposed by Thiele et al. [34], because a one-sided consideration of an advantageous or disadvantageous effect is less promising [35]. While every nutrient can have beneficial or adverse effects, the nutriRECIPE-Index includes components with a minimum and maximum target value, considering current recommendations and nutrition patterns. The reference values for macro- and micronutrients of the German Nutrition Society (DGE), the Austrian Nutrition Society (ÖGE) and the Swiss Nutrition Society (SGE) [36, 37] served as references. The reference value for protein was modified in line with more recent studies that found more beneficial effects when protein was ingested in amounts of 1.2 g per kg body weight per day [38–41]. The German Nutritional Society (DGE) and the World Health Organization (WHO) have specified the minimum recommended protein intake as 0.8 g/kg body weight (BW) [36, 42], but the suggested optimal protein intake of 1.2 g/kg BW is used as the basis for calculating the nutriRECIPE-Index. It is also important to note that lunch is often the most protein-rich meal of the day. The maximum sugar content per meal follows the recommendation of the WHO [43], which corresponds with recommendations of the DGE’s consensus paper on sugar consumption [44].

**Table 1:** Nutrients integrated into the nutriRECIPE-Index, their target values and weighting factors. Requirement values for macronutrients and micronutrients according to recommendations of DGE and WHO, Values for Bioactive Plant Compounds are calculated based on DGE-meal plans for 1 week, which incorporate the “Five-A-Day” recommendation for fruits and vegetables

	Nutrient	Daily recommendation	Unit	Average intake/day	Weighting factor
**Favourable nutrients**					
Carbohydrates	Fibre	30	g	25.70	1.25
Proteins	Protein	84	g	78.75	1.07
Fats	Mono- and poly-unsaturated fatty acids	20	% of energy	20.00	1.00
Vitamins	Vitamin D	20	μg	3.35	3.00
	Vitamin E	14	mg	14.70	0.95
	Thiamine	1,2	mg	1.55	0.77
	Riboflavin	1,4	mg	1.95	0.72
	Vitamin B6	1,5	mg	2.30	0.65
	Folic acid	300	μg	314.00	0.96
	Vitamin B12	4	μg	5.40	0.74
	Vitamin C	110	mg	152.00	0.72
Minerals	Calcium	1000	mg	1081.00	0.93
	Magnesium	350	mg	412.50	0.85
	Iron	15	mg	13.75	1.09
	Iodine	200	μg	102.50	1.95
	Zinc	10	mg	10.90	0.92
**Unfavourable nutrients**					
Carbohydrates	Added sugars	50	g	92.00	1.84
Fatty acids	Saturated fatty acids	10	% of energy	10.00	1.00
Minerals	Salt (sodium)	6	g	7.44	1.24
**Bioactive Plant Compounds**					
	Carotenoids	20	mg	5–6	1.00
	Glucosinolates	40	mg	< 50	1.00
	Phenolic Acids	200	mg	200–300	1.00
	Polyphenols	300	mg	100–150	1.00
	Phytosterols	450	mg	170–440	1.00

While other indices (e.g., the Healthy Eating Index or the Menu Sustainability Index) usually assume linear correlations between the level of nutrient implementation and the health effect, the nutriRECIPE-Index is based on the concept of *diminishing marginal utility* and the logarithmic relationship of Bernoulli [45]. Moreover, the degree of fulfilment is not observed in absolute terms but is related to the energy content of the menu, so the nutrient density is evaluated as a result. **Supplemental file** **2** in the supplementary material shows how the results differ when analysing the amount of nutrients in a recipe considering its energy content, in comparison to just assuming that a menu should include one-third of the amount of nutrients in a daily recommendation.

To calculate the degrees of fulfilment, the following formulas are used:

For favourable nutrients: f(x) = ln(x) + 1.

For unfavourable nutrients: f(x) =  − ln(x).

If the density of a favourable nutrient is high enough that the menu includes the daily requirement of that nutrient, the function obtains the value “1”. If the maximum recommended daily intake of an unfavourable nutrient is not exceeded, the function obtains the value “0”. However, if the density for a specific nutrient is tripled so that one-third of the energy is already sufficient to cover the daily requirement, the logarithmic function returns the value “2.1”, which is also the unweighted maximum value for favourable nutrients (upper cut-off). On the other hand, exceeding the recommended daily amounts of unfavourable nutrients leads to malus points, whereby the unweighted minimum value is “-2.1” (lower cut-off).

According to Waijers et al. [25], de facto nonweighting of the various compounds of a score is also a weighting, namely, where all components are weighted equally. To avoid this bias, the nutriRECIPE-Index applies a moderate weighting taking into account the degree of supply within the target group (in this publication, the population of Germany) based on officially documented intake data from the last nutrition survey in Germany [46] and based on the method of ecological scarcity [47]. The more inadequate the supply is in the overall population with regard to the nutrient, the higher the weighting of the nutrient and the higher the impact in the assessment. On the other hand, if the supply in the general population is in accordance with the corresponding recommendation, the weighting factor is 1. At the same time, the supply level of the nutrient supply can change the number of bonus points by multiplying the unweighted maximum value of “2.1” or the minimum value of “-2.1” by the corresponding weighting factor for the particular nutrient. Table 1 gives an overview of the weighting factors used.

The formula for the nutriRECIPE scores for nutrients with beneficial or adverse effects is as follows:

$$ \boldsymbol{y}=\left(\boldsymbol{ln}\left(\frac{\boldsymbol{Nmenu}}{\boldsymbol{Nrec}}\ast \frac{\boldsymbol{Erec}}{\boldsymbol{Emenu}}\right)+\mathbf{1}\right)\ast \frac{\boldsymbol{Nrec}}{\boldsymbol{Nact}} $$

$$ \boldsymbol{y}=\left(-\boldsymbol{ln}\ \left(\frac{\boldsymbol{Nmenu}}{\boldsymbol{Nrec}}\ast \frac{\boldsymbol{Erec}}{\boldsymbol{Emenu}}\right)\right)\ast \frac{\boldsymbol{Nrec}}{\boldsymbol{Nact}} $$

N_menu_ – nutrient content in a menu

N_rec_– recommended nutrient intake per day

E_rec_ – recommended energy intake per day

E_Menu_ – energy content of one menu

N_act_ – average nutrient intake per day

The sum of the single nutrient scores results in the nutriRECIPE-Index. The higher the index value is, the more nutrients there are contained in the dish in a balanced ratio. Given the weightings, a total score of 100% is possible, even if not all the respective dietary target values are met. With bonus points, it is also possible for a menu (e.g., if the nutrient reference values are exceeded) to have a nutriRECIPE-Index higher than 100%.

#### Considering bioactive plant compounds

A particular focus during the index development was the inclusion of BPCs. As no food-specific reference databases exist, a separate database has been created containing information on the occurrence and content of BPCs in fruits, vegetables, nuts and oil seeds [48]. The eBASIS database [49] served as a basis. Data for phytosterols in bread and cereals were added from Normen et al. [50] Retention factors of BPCs for different cooking methods were taken from the review of Palermo et al. [51] and for phytosterols from Thanh et al. [52] The nutriRECIPE score ultimately includes aggregated sum values for the five main classes of BPCs: carotenoids, phenolic acids, polyphenols, glucosinolates, and phytosterols.

#### Recipe origin and validation

Using the nutriRECIPE-Index, a complete 6-week menu cycle of a university canteen (Chemnitz in Saxony, Germany) was evaluated, allowing adjustments to the model to review its usability. This menu included standard and MensaVital recipes. MensaVital® is a trademark used for certain dishes in German student canteens that claim to be physiologically balanced [53]. Validation was carried out by comparing the results of the analysis of the recipes using two established and validated tools for the assessment of nutritionally balanced meals in canteens, the Healthy Meals Index [18] and the Nutri-Score [19].

#### NutriRECIPE database

For effective data acquisition and processing, the recipes were recorded in a self-generated MS Access® database. The data of the German Nutrition Database Federal Food Code (BLS) Version 3.02 and the data of the BPC database were stored and linked via the BLS code number.

## Results

**Fig. 1:**
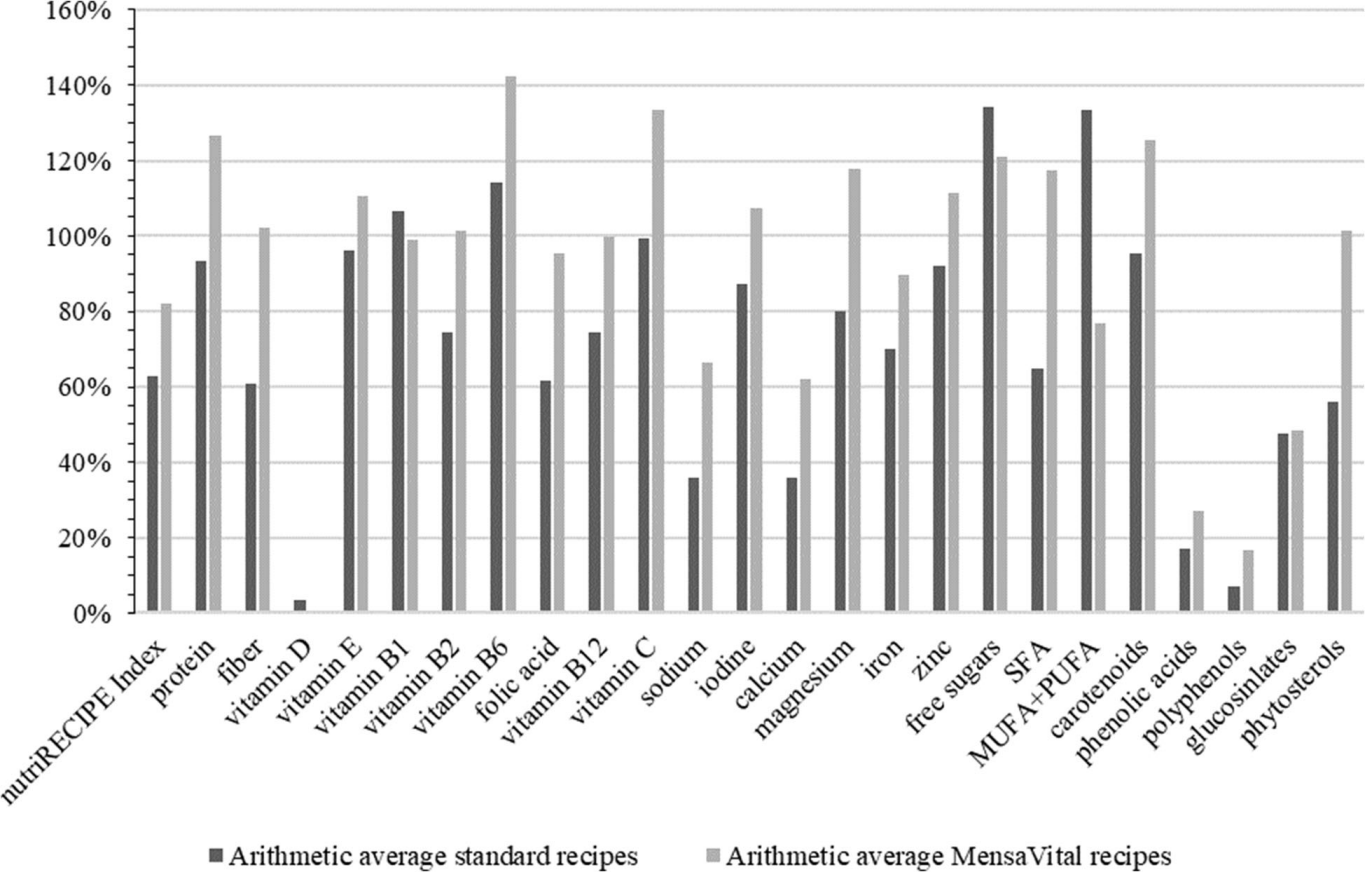
Means of single nutriRECIPE nutrient scores of the menu cycle. A value of 100% for a favourable nutrient means that the average content of all recipes contains precisely the amount required to meet the recommendations of the D-A-CH reference values. In the case of unfavourable nutrients, a value of 100% corresponds to the fact that the maximum recommended intake was strictly followed

The results of the recipe evaluation using the nutriRECIPE-Index are shown in Fig. 1 as well as in **Supplemental file** **1**, **Supplemental file** 3 and **Supplemental file** **4**. The arithmetic mean of the nutriRECIPE-Index of all 106 individual recipes is 65.2% [confidence interval (CI) 95%: 61.6–68.8%]. The lowest value observed was 16.8%, and the highest value calculated was 120.8% due to bonus points. A separate evaluation, distinguishing between standard recipes (93) and MensaVital® recipes [13], resulted in higher mean and median values for MensaVital® (arithmetic mean of standard recipes: 62.8% [CI 95%: 59.1%; 66.6%], MensaVital®: 82.2% [CI 95%: 75.3%; 89.0%]).

Figure 1 and **Supplemental file** **3** show an evaluation of the 24 individual nutrients and nutrient groups included in each dish’s nutriRECIPE score. A value of 100% for a favourable nutrient means that the average content of all foods (Fig. 1 arithmetic mean; **Supplemental file** **3** median values) contains exactly the amount of that nutrient required to meet the recommendations of the D-A-CH reference values. In the case of unfavourable nutrients, a value of 100% corresponds to strict adherence to the maximum recommended daily intake.

The protein assessment for both menu lines was above 90%. Higher values of the MensaVital® recipes were reached because they contain significantly fewer calories (759 kcal) than the standard recipes (996 kcal) with a similar amount of protein. For dietary fibre, the recommendations for the standard recipes are only partially fulfilled, with an average of 60.7% [CI 95%: 53.4%; 68.0%]. MensaVital® recipes with mean values of 102.0% [CI 9%: 85.1%; 118.9%] achieved dietary fibre scores according to the recommendations.

The supply of vitamins E, C, and B is between 60 and 90% for standard recipes. The MensaVital® recipes show all values above 90% (see Fig. 1). Especially for vitamin B2 and folate, the standard recipes dropped to mean values of 74.4% [CI 95%: 65.0%; 83.8%] and 61.8% [CI 95%: 53.5%; 70.0%], clearly below the values of the MensaVital® recipes (vitamin B2: 101.6% [CI 95%: 77.5%; 125.6%]; folate: 95.4% [CI 95%: 75.7%; 115.1%]). As expected, the menus did not contribute to an adequate vitamin D supply, as the values were 0% for almost all analysed dishes.

The supply of minerals is comparable to that of vitamins. In standard recipes, the nutriRECIPE nutrient scores for iodine, magnesium, and zinc were consistently above 75% on arithmetic average and above 60% for iron. With MensaVital® recipes, the scores for iodine, magnesium, and zinc were above 95%, and for iron, the score was above 75% (see Fig. 1). The calcium supply was insufficient in both menu lines, so the nutriRECIPE nutrient score for standard recipes arithmetically averages 35.8% [CI 95%: 26.1%; 45.6%] and for MensaVital® recipes 62.1% [CI 95%: 35.8%; 88.4%].

In the case of unfavourable nutrients, sodium intake in both menu lines was markedly higher than recommended, whereby the recommendations for MensaVital,® with a score of 66.3% [CI 95%: 33.1%; 99.4%], were better fulfilled compared to the standard recipes, with a score of 36.0% [CI 95%: 25.7%; 46.2%]. The standard recipes contained more saturated fatty acids than recommended so that the score reached values of 65.0% [CI 95%: 59.1%; 70.8%]. The total sugar content in both recipe lines was not problematic for most menus. However, the two standard dishes “semolina porridge with sour cherries” and “rice pudding with applesauce” exceeded the daily WHO recommendations for sugar by almost three times.

The MensaVital® recipes were characterised by higher contents of BPCs than the standard recipes. Because lunchtime meals usually contain little fruit, the two main classes of phenolic acids and polyphenols, which are mainly found in fruit, are present in significantly lower amounts than carotenoids, glucosinolates, and phytosterols (see Fig. 1).

### Validation

The first validation was performed using the food group and macronutrient-based Healthy Meal Index [18], comprising the three items: “fruits and vegetables”, “fat quantity and quality”, and “whole grains and potatoes”. According to the Healthy Meal Index, a dish receives points ranging from “0” to “6” (see Table 2).

**Table 2:** Evaluation scheme according to the Healthy Meal Index [18] (simplified)

Compound	Rating	Criteria	Remarks
fruits and vegetables	0	< 1 unit	< 75 g
	1	> = 1 unit	> = 75 g
	2	> 2 units	> = 150 g
fat quantity and quality	0	amount fat units > amount starch units	fat unit: 5 g; starch unit: > = 400j = 23,5 g carbohydrates
	1	amount fat units = amount starch units	(minus sugar and sweets)
	2	amount fat units < amount starch units	or: amount fat units = amount starch units and the fat is from plants
wholegrain and potatoes	0	< 0,5 units wholegrain/potatoes	< 37,5 g pasta/rice or 25 g bread or 75 g potatoes
	1	> = 0,5 units wholegrain/potatoes	> = 37,5 g pasta/rice or 25 g bread or 75 g potatoes
	2	> = 1 units wholegrain/potatoes	> = 37,5 g pasta/rice or 25 g bread or 75 g potatoes

**Fig. 2:**
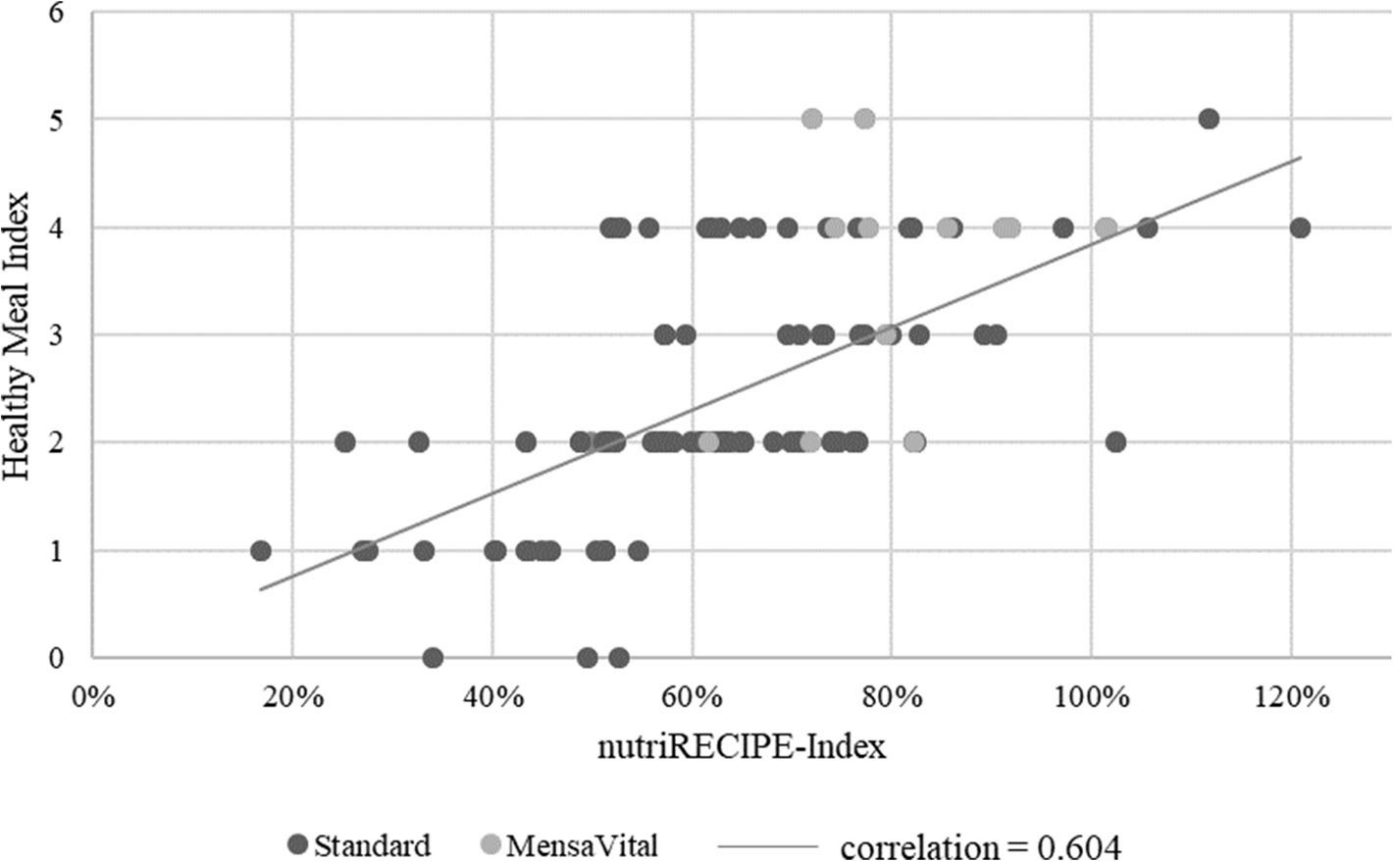
Correlation of nutriRECIPE and Healthy Meal Index [18] scores of the menu cycle (106 recipes of a university canteen, Germany)

The Healthy Meal Index suggests, as seen in Table 2, one out of two points if the amounts of fat and starch are equal. In the following analysis, a deviation of 25% in the difference between carbohydrates and fat is tolerated, and in that case, the two are considered equal. Otherwise, the more precise carbohydrate and fat quantities of the nutritional analysis are used for calculation instead of the simplified calculation according to food groups by Lassen et al. [18] Figure 2 illustrates the correlation of the scores of the 6-week menu cycle (divided into standard and MensaVital recipes) determined by the nutriRECIPE-Index and the Healthy Meal Index, which has a correlation coefficient of r = 0.604.

As shown, high nutriRECIPE scores are correlated with high Healthy Meal Index scores and vice versa. The best-rated dish according to the Healthy Meal Index (HMI value of 6 = 100%), that is, “vegetable plate with Dutch sauce” and potatoes, received a nutriRECIPE score of 24.9 = 112%. The recipe with the highest nutriRECIPE score of 26.9 = 120.8% is only rated as mediocre by the Healthy Meal Index (4 = 66.66%). After applying both methods as the basis for calculations, the MensaVital® menu line reached higher values on average than the standard menu line. However, because the correlation was only 0.604, there were some deviations. The differences can be explained, on the one hand, by the focus of the HMI on only three criteria, which are therefore very important, and on the other hand, by a low differentiation within the categories compared to the nutriRECIPE-Index. The vast majority (84%) of the dishes were high in fat and received zero points in the fat content category. While 46% of the MensaVital® recipes received one or two points in the fat content category, only 2% of the standard recipes received more than zero points. Furthermore, less than 10% of all dishes failed to receive the maximum possible value of 2 points in the fruit and vegetable category. This could also indicate the cultural specificity of the HMI because the quantities of fruits and vegetables consumed in Scandinavian countries are generally lower than those in southern European countries (although these differences are getting smaller) [54]. It would be desirable to produce a more pronounced differentiation similar to the evaluation of the dishes considered here.

The second validation was performed using the Nutri-Score, which is used as a front-of-package label in Europe, particularly in France [19]. The Nutri-Score is food- and nutrient-based, comprising seven items. In general, the Nutri-Score first calculates points, which are split into five different point ranges with a letter code from “A” to “E”. For an additional illustration, the background of the letter code has traffic light colours. The unfavourable food components included in this calculation are calories, total sugars, saturated fatty acids, and sodium. As favourable food components, protein and dietary fibre, as well as the portions of fruits, vegetables, and nuts, are evaluated. All calculations performed are based on 100 g with defined limits. There are specific adjustments for beverages, cheese, and fatty spreads such as margarine [19]. The lower the Nutri-Score, the better is the nutritional profile of the food or drink.

**Fig. 3:**
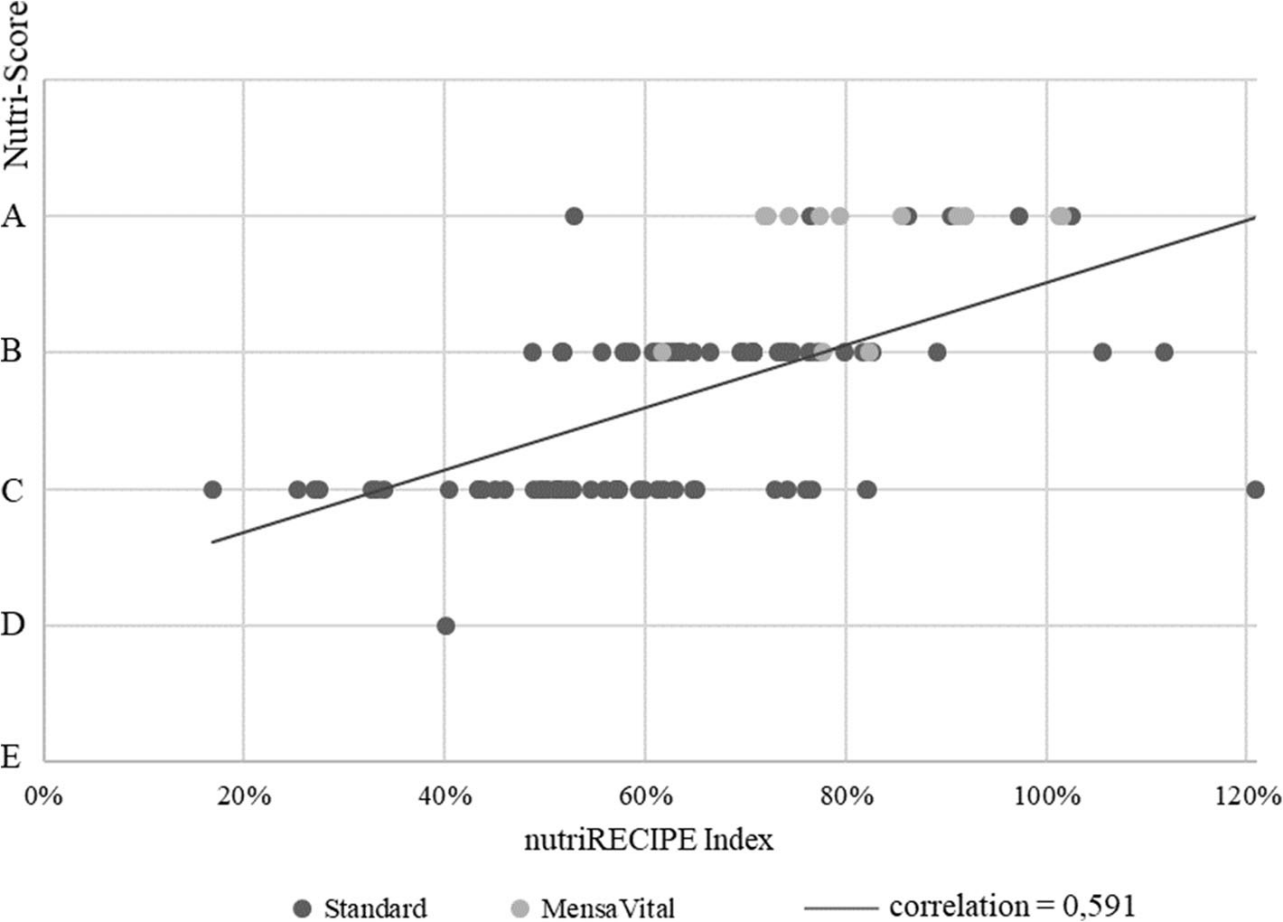
Correlation of the nutriRECIPE-Index and the Nutri-Scores [19] of the meal cycle (106 recipes of the university canteen, Germany)

Figure 3 shows that high values of the nutriRECIPE-Index correlate with low values of the Nutri-Score (inverse plot). The majority of MensaVital® dishes received an A rating, and a few received a B rating. The standard dishes range from A to D, with the majority of the dishes rated C. The coefficient for the correlation of Nutri-Score and nutriRECIPE-Index is 0.591.

Although the evaluation results of both indices seem similar, a closer look reveals the weaknesses of the relatively simple Nutri-Score. The Nutri-Score does not adequately assess the two worst dishes according to the nutriRECIPE-Index, “semolina with cherries” (16.8%) and “rice pudding with applesauce” (25.4%). Both dishes receive a C rating, although they contain 164 g and 128 g of sugar, respectively, per serving. Both dishes contain no vegetables, very little dietary fibre, and only small amounts of micronutrients because canned fruits are not an adequate replacement for vegetables.

The assumption that low sugar and fat contents and high protein and fibre contents may lead to a sufficient micronutrient supply is likewise disproved several times. For example, the dish “BBQ Chicken” receives an A-rating with the Nutri-Score. Taking the nutriRECIPE-Index as a basis, the rating of “BBQ Chicken” is only 53%. This difference is caused by the fact that the nutriRECIPE-Index includes micronutrients and BPCs.

## Discussion

Here, we successfully developed a novel food quality assessment tool, the nutriRECIPE-Index, which has several strengths and innovative features:

1. Consideration of 19 macro- and micronutrients with an official recommendation status,
2. Weighting of all considered nutrients based on representative supply data,
3. Inclusion and assessment of bioactive plant compounds.

With its nutrient-based approach, the nutriRECIPE-Index is universally applicable and can thus facilitate adherence to a healthy diet in the population despite cultural diversity and individual taste preferences. Similarly, it may be easier for policymakers to recommend healthy diets when not solely linked to specific foods. For example, aspects of vegetarian nutrition, such as avoiding meat or fish, are often generally devalued in other indices (see the Healthy Eating Index or Mediterranean Diet Score) but not within the nutriRECIPE-Index. Adjustments at the recipe level are a further advantage compared to other indices that measure nutritional behaviour over a long period. Although it is evident that single meals have only a tiny impact on overall diet quality, people make their choices at this level, and the sum of these single choices constitutes healthy eating behaviour. Additionally, by referring to the energy value of the menus to be evaluated, it is possible to adjust the quantities individually and therefore enable population subgroup-specific or even personalised nutrition. Existing indices, such as the ONQI or the Healthy Eating Index, systematically disadvantage low-calorie meals. Although not applied in this study, the reference values of nutrients within the nutriRECIPE-Index can prospectively be adapted to the needs of different target groups, such as older people, children or physically active people.

The weighting of the nutrients within the nutriRECIPE-Index should be seen as an additional feature that can be applied where reliable supply data of the target population are available. We understand that this might not be the case for many countries in the world. However, for Germany, it is a valuable option to further tailor the nutriRECIPE-Index to the needs of the target population. For example, Germany is an iodine-deficient area. Therefore, iodine has a high weighting factor (see Table 1) within the nutriRECIPE-Index, whereas the German population is more than well supplied with vitamin C. In the final result, an adequate iodine supply has an almost three times greater influence on the nutriRECIPE-Index than an adequate vitamin C supply. Due to the unique properties of the logarithm function within the nutriRECIPE-Index, nutrients with high weighting factors can also receive more extra points (see the Methods section) than nutrients with low weighting factors.

Furthermore, the integration of bioactive plant compounds (BPCs) into the nutriRECIPE-Index is thus far unique in the field of nutrition indices. At the same time, the difficulty here lies in the fact that it has not been possible to access established systems and databases. First, the concentrations of BPCs in plants vary markedly. Second, the role of food processing in bioavailability cannot be fully considered due to a lack of data. Investigations on the content of BPCs and their modification during processing are still incomplete and should be improved in the future. In addition, it cannot be assumed that all BPCs contained in our edible plants have already been found and characterised. Here, future studies will complete the mosaic with ever-advancing analytical methods. For these reasons, the amount of BPCs is integrated as the sum of the five main classes (carotenoids, phenolic acids, polyphenols, glucosinolates, and phytosterols) and has been included as one score in the nutriRECIPE-Index with no differentiated subdivision according to subclasses or even individual compounds. Our calculations show that lunch menus usually contain a maximum of three of these five main classes. Thus, with these extra points, the importance of large portions of vegetables, fruits, legumes, nuts, and vegetable oils in nutrition is emphasised without overestimating the micronutrients.

Challenges and limitations in the application of the nutriRECIPE-Index arise mainly from the availability of high-quality data. The first and most important is the availability of an up-to-date and detailed nutrient database. Constant improvement in nutrient databases is significant to strengthen nutrition indices. Furthermore, exact recipes are necessary for nutrient-based analyses. However, in catering facilities, such recipes are not always available, and some ingredients, such as salt, oil and spices, are not precisely measured. A fundamental problem with using reference values for nutrients is that individual needs for nutrients may differ. Moreover, possible interactions between the nutrients themselves and between nutrients and the food matrix are too complex to be considered. However, we have to stress that the last two points are not specific to the nutriRECIPE-Index.

Finally, there are other aspects of healthy eating behaviour: food quality and availability, actual recipe preparation, nutritional culture, and social desirability, which can hardly be incorporated into nutrition indices. It would be advisable to validate health outcomes through human trials in the future to obtain more accurate information on the efficacy of a priori indices.

## Conclusion

The nutriRECIPE-Index allows an extensive evaluation and optimisation of complex dishes, considering 24 nutrients and nutrient groups plus supply-dependent weighting factors. The nutriRECIPE-Index permits structural adjustments, e.g., concerning the nutrients included or the weighting depending on the supply status of the population group under consideration. Therefore, adapting to individual target groups such as older people, children, and physically active people is possible and desirable. Through these adaptations, other reference values can be applied, and different supply situations can be addressed. Thus, a nutrient-specific assessment is possible, representing a decisive advantage of the nutriRECIPE-Index over other general nutrition indices. The nutriRECIPE-Index offers the opportunity for caterers to optimise their menus, which in turn can improve public health.

## Supplementary Information

**Supplemental file 1. Table S1:** Key figures for recipe evaluations with the nutriRECIPE-Index.

**Supplemental file 2. Figure S1:** Non-linear scaling of a nutrient score depending on the degree of fulfilment of the recommendation in the nutriRECIPE-Index, with the x-axis representing the degree of fulfilment of the recommendation and the y-axis representing the unweighted nutriRECIPE score.

**Supplemental file 3. Figure S2:** Median values of single nutriRECIPE nutrient scores for the menu cycle. A value of 100% for a favourable nutrient means that the average content of all recipes contains precisely the amount required to meet the recommendations of the D-A-CH reference values. In the case of unfavourable nutrients, a value of 100% corresponds to the fact that the maximum recommended intake was strictly followed.

**Supplemental file 4. Figure S3:** Overview of all nutriRECIPE-Index values of the menu cycle.

## Data Availability

All data generated or analysed during this study are included in this published article and its supplementary information files. Any additional information on the datasets used and/or analysed during the current study, are available from the corresponding author on reasonable request.
