# Correction: The nutriRECIPE-Index– development and validation of a nutrient-weighted index for the evaluation of recipes

**DOI:** 10.1186/s40795-025-01061-x

**Published:** 2025-07-15

**Authors:** Frank Forner, Ina Volkhardt, Toni Meier, Olaf Christen, Gabriele I. Stangl

**Affiliations:** 1 Martin-Luther-University Halle-Wittenberg, Institute for Agricultural and Nutritional Sciences, Halle, Germany; 2 NutriCARD Competence Cluster for Nutrition and Cardiovascular Health, Halle-Jena Leipzig, Leipzig, Germany

**Correction: BMC Nutr 7, 74 (2021)**

10.1186/s40795-021-00483-7

Following publication of the original article [1], the authors identified an error in the formula for the nutriRECIPE scores for nutrients within the *Methods* section of the article.

The incorrect formulae is:

$$\:\varvec{y}=\left(\varvec{ln}\left(\frac{\varvec{N}\varvec{m}\varvec{e}\varvec{n}\varvec{u}}{\varvec{N}\varvec{r}\varvec{e}\varvec{c}}\varvec{*}\frac{\varvec{E}\varvec{r}\varvec{e}\varvec{c}}{\varvec{E}\varvec{m}\varvec{e}\varvec{n}\varvec{u}}\right)+1\right)\varvec{*}\frac{\varvec{N}\varvec{a}\varvec{c}\varvec{t}}{\varvec{N}\varvec{r}\varvec{e}\varvec{c}}$$

$$\:\varvec{y}=\left(-\varvec{l}\varvec{n}\:\left(\frac{\varvec{N}\varvec{m}\varvec{e}\varvec{n}\varvec{u}}{\varvec{N}\varvec{r}\varvec{e}\varvec{c}}\varvec{*}\frac{\varvec{E}\varvec{r}\varvec{e}\varvec{c}}{\varvec{E}\varvec{m}\varvec{e}\varvec{n}\varvec{u}}\right)\right)\varvec{*}\frac{\varvec{N}\varvec{a}\varvec{c}\varvec{t}}{\varvec{N}\varvec{r}\varvec{e}\varvec{c}}$$

The correct formulae is:

$$\:\varvec{y}=\left(\varvec{ln}\left(\frac{\varvec{N}\varvec{m}\varvec{e}\varvec{n}\varvec{u}}{\varvec{N}\varvec{r}\varvec{e}\varvec{c}}\varvec{*}\frac{\varvec{E}\varvec{r}\varvec{e}\varvec{c}}{\varvec{E}\varvec{m}\varvec{e}\varvec{n}\varvec{u}}\right)+1\right)\varvec{*}\frac{\varvec{N}\varvec{r}\varvec{e}\varvec{c}}{\varvec{N}\varvec{a}\varvec{c}\varvec{t}}$$

$$\:\varvec{y}=\left(-\varvec{l}\varvec{n}\:\left(\frac{\varvec{N}\varvec{m}\varvec{e}\varvec{n}\varvec{u}}{\varvec{N}\varvec{r}\varvec{e}\varvec{c}}\varvec{*}\frac{\varvec{E}\varvec{r}\varvec{e}\varvec{c}}{\varvec{E}\varvec{m}\varvec{e}\varvec{n}\varvec{u}}\right)\right)\varvec{*}\frac{\varvec{N}\varvec{r}\varvec{e}\varvec{c}}{\varvec{N}\varvec{a}\varvec{c}\varvec{t}}$$

The original article [1] has been updated.
