# Supplementary figures and images for: The nutriRECIPE-Index – development and validation of a nutrient-weighted index for the evaluation of recipes
Source: BMC Nutr. 2021 Nov 18;7:74. doi: 10.1186/s40795-021-00483-7 (PMC8600763; doi:10.1186/s40795-021-00483-7)

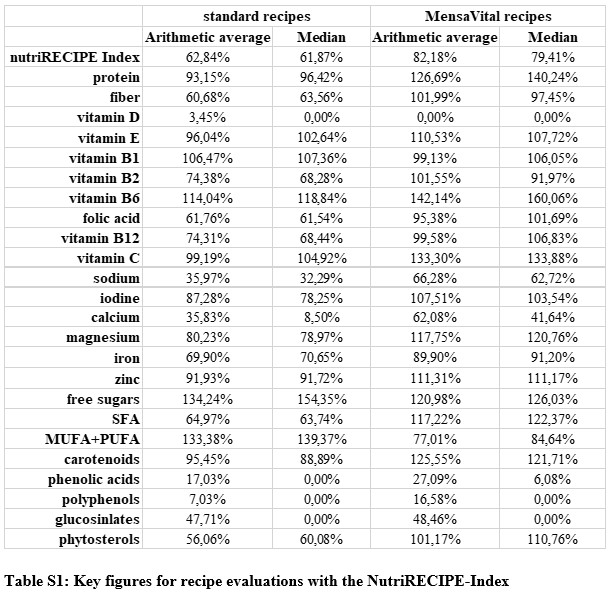

Supplement: Supplementary file 1 — Supplemental file 1. Table S1: Key figures for recipe evaluations with the nutriRECIPE-Index. [file 40795_2021_483_MOESM1_ESM.jpg]

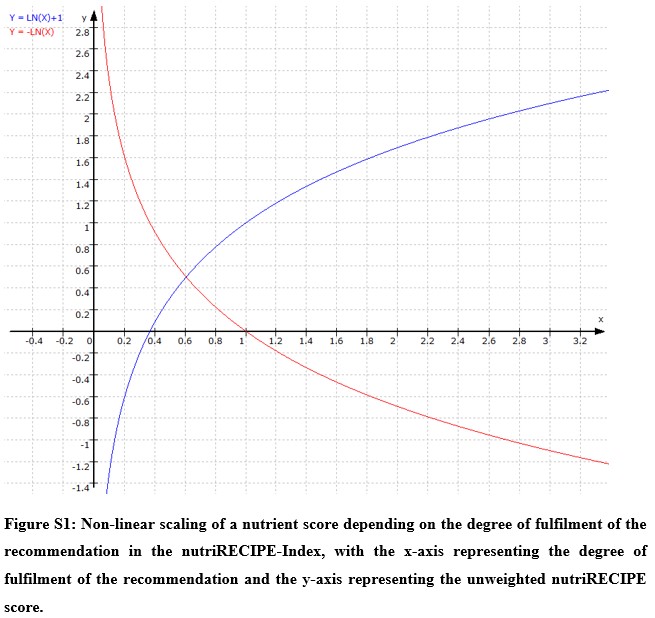

Supplement: Supplementary file 2 — Supplemental file 2. Figure S1: Non-linear scaling of a nutrient score depending on the degree of fulfilment of the recommendation in the nutriRECIPE-Index, with the x-axis representing the degree of fulfilment of the recommendation and the y-axis representing the unweighted nutriRECIPE score. [file 40795_2021_483_MOESM2_ESM.jpg]

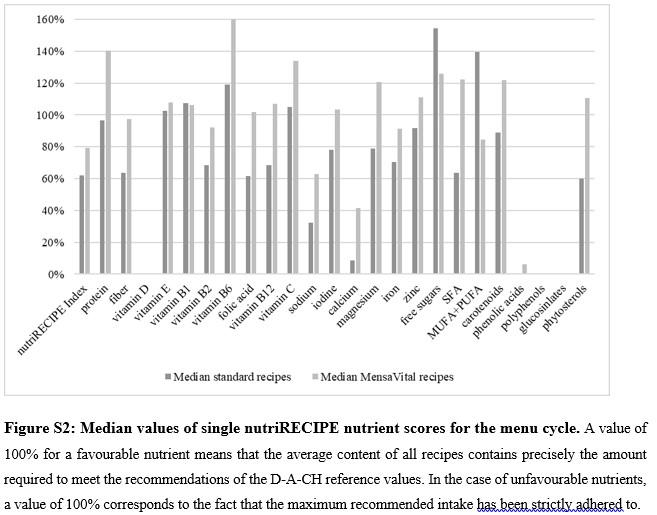

Supplement: Supplementary file 3 — Supplemental file 3. Figure S2: Median values of single nutriRECIPE nutrient scores for the menu cycle. A value of 100% for a favourable nutrient means that the average content of all recipes contains precisely the amount required to meet the recommendations of the D-A-CH reference values. In the case of unfavourable nutrients, a value of 100% corresponds to the fact that the maximum recommended intake was strictly followed. [file 40795_2021_483_MOESM3_ESM.jpg]

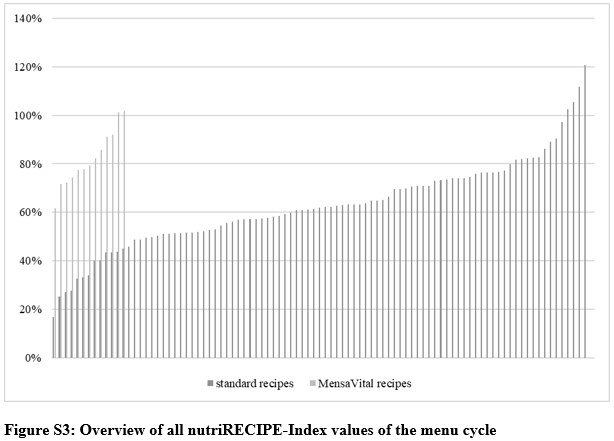

Supplement: Supplementary file 4 — Supplemental file 4. Figure S3: Overview of all nutriRECIPE-Index values of the menu cycle. [file 40795_2021_483_MOESM4_ESM.jpg]
